# High-Moisture Extrusion of Plant-Based Meat Analogues: Challenges and Opportunities

**DOI:** 10.3390/foods15193388

**Published:** 2026-09-23

**Authors:** Sanjaya Karki, Rishabh More, Edward Attenborough, Sushil Dhital

**Affiliations:** 1 Bioresource Processing Research Institute of Australia (BioPRIA), Department of Chemical and Biological Engineering, Monash University, Clayton, VIC 3800, Australia; 2 Department of Chemical and Biological Engineering, Monash University, Clayton, VIC 3800, Australia

**Keywords:** extrusion, plant-based meat analogue, peptidomics, extrusion processing parameters

## Abstract

Growing concerns about the environmental, ethical, and public health impacts of conventional meat production have driven the development of plant-based meat analogues (PBMAs). Among the plant protein structuring techniques available, high-moisture extrusion (HME) is one of the most scalable structuring technologies for producing fibrous, anisotropic textures that mimic the organization of animal muscle. However, achieving meat-like characteristics remains challenging due to the inherent differences between plant proteins and animal muscle proteins, as well as the complex interactions between formulation and processing conditions that determine final product structure. Therefore, this review provides an assessment of extrusion-based PBMA development by integrating current understanding of protein transformations during extrusion, including critical processing parameters such as moisture content, temperature profile, screw configuration, screw speed, and cooling die design. The key challenges that limit the wider adoption of PBMAs, such as structural, sensory, nutritional, and processing challenges, are critically discussed. Furthermore, emerging opportunities for the development of PBMAs, such as advanced formulation, hybrid processing, or the use of analytical tools such as peptidomics, are highlighted. Overall, this narrative review summarizes the current state of extrusion-based PBMA development and highlights the key scientific and technological advances needed to develop plant-based products that more closely replicate the structural, sensory, and nutritional qualities of animal meat while maintaining economic and environmental sustainability.

## 1. Introduction

The global food system faces unprecedented challenges in meeting protein demands while addressing environmental sustainability and ethical concerns. Some projections suggest that the conventional meat-to-alternative food consumption ratio is expected to decline from 9:1 in 2025 to 4:6 by 2040, largely driven by environmental concerns over the excessive use of land and water resources and its adverse impact on terrestrial and aquatic biodiversity [1,2,3,4,5,6]. However, the extent of these environmental impacts varies across production systems and assessment methodologies. Despite the projected growth in alternative foods, demand for animal protein remains high and contributes to the expansion of livestock production and associated environmental concerns, while animal-based foods have been estimated to account for approximately 57% of GHG emissions across the entire food system [7,8]. Alongside the environmental concerns, current dietary patterns also contribute significantly to public health burdens. In the European Union alone, the hidden public health costs associated with poor diets are estimated at nearly €900 billion annually, with underconsumption of legumes, wholegrains, and vegetables serving as a key driver of cardiovascular diseases and type II diabetes [9]. As a combined result of growing awareness of animal welfare, sustainability concerns, public health and the rise in protein demand, there has been considerable interest in replacing traditional animal protein sources, such as meat, seafood, and dairy products, with next-generation plant-based protein sources that can mimic their properties [10,11]. Accordingly, adoption of plant-based protein products such as plant-based meat analogues is a step towards building a more sustainable, ethical and resilient food system.

Plant proteins such as soy, wheat gluten, and pea proteins are commonly used raw materials to achieve desirable textural and fibrous structures through extrusion, while alternative sources such as lentil, lupin, chickpea, cottonseed, peanut, rapeseed, and sesame are emerging as promising ingredients [12,13,14,15,16,17]. However, plant proteins often lack the structural and sensory characteristics of animal meat, particularly in terms of texture and fibrous structure. Most plant proteins have globular structures and exist as complex multimers held together by physical or chemical bonds [18,19], posing challenges for texturization in meat analogues. Therefore, appropriate processing is required to enhance the techno-functional performance of plant proteins for the development of plant-based meat analogues (PBMAs).

The acceptance of PBMAs depends critically on how closely they mimic the properties of conventional meat, such as the appearance, textural properties, juiciness, and sensory experiences derived from animal meat [3,20,21]. Mimicking the complex fibrous and anisotropic structure of animal muscle with plant-based proteins remains a major challenge that limits the wider adoption of plant-based meat alternatives [21,22]. As a result, various ingredients (proteins, polysaccharides, fibers, salts, lipids, etc.) and processing techniques play a crucial role in mimicking the properties of meat. Several structuring technologies have been developed for the creation of plant-based meat-like structures. The most current technologies used for the development of plant-based meat analogues include electrospinning, thermo-extrusion, shearing, freeze structuring, and 3D printing [23,24,25,26,27]. However, only a few methods are currently scalable due to a high cost-to-production ratio. Among them, extrusion—a continuous process that can be easily scaled up—is widely used for PBMA production.

Extrusion is a commonly used technique for restructuring plant-based materials into fibrous products. Commonly, extrusion is practiced in two ways: low-moisture extrusion and high-moisture extrusion [12,25,28,29]. Low-moisture extrusion (LME) uses less than 30% moisture to produce texturized vegetable protein (TVP) with a meat-like structure after rehydration, while high-moisture extrusion (HME) uses more than 50% moisture to create fibrous, whole meat analogues through protein alignment and intermolecular bonding [30]. HME is used more commonly than other methods for the production of fibrous textures superior to texturized vegetable proteins. It effectively combines heat, shear forces and moisture to denature proteins and align them into anisotropic structures resembling animal muscle fiber, which often requires optimization of parameters such as temperature, screw speed, moisture content and die geometry to achieve desirable textural properties consistently [21,31].

Despite industrial relevance, the application of extrusion remains largely a black box because the complex physicochemical and structural transformations inside the extruder are difficult to monitor directly [32,33]. The structuring of plant proteins presents several technical and market challenges. These include consumer perception of PBMAs, variability in the functionality of raw materials, limited understanding of protein–protein and protein–polysaccharide interactions during processing, and inconsistencies in the texture and sensory quality of final products [10,34]. In addition, HME is associated with various variables (temperature, screw speed, moisture content, screw configuration, die geometry and protein source) and high energy consumption, which can restrict the scalability of PBMAs. Furthermore, scaling up HME remains challenging because the processing itself is not linear between the laboratory, pilot and industrial scales, as changes in extruder dimensions and operating conditions can alter heat transfer, flow behavior, residence time, and shear distribution, resulting in variations in protein restructuring and final product quality. Nevertheless, significant opportunities exist for advancing next-generation PBMAs through the exploration of alternative plant protein sources, extrusion parameter optimization, and process or raw material modification techniques. Accordingly, raw material and processing conditions should be carefully designed to overcome the technical and market challenges.

Existing reviews have primarily discussed PBMA development from the perspectives of ingredient selection, nutritional aspects, sensory challenges, or general processing approaches. For example, Das et al. [14] provided an overview of HME for PBMAs but did not extensively evaluate critical processing parameters and opportunities for improving extrusion-based products. Kurek et al. [16] provided a general discussion of HME rather than a detailed analysis of extrusion parameters for PBMA processing. In contrast, this narrative review provides a critical evaluation of HME with emphasis on the processing parameters. Importantly, it provides a framework for challenges across various dimensions, such as textural and structural limitations, sensory defects, nutritional inadequacies, and processing-related concerns, increasing our understanding of barriers to achieving meat-like products. Also, promising opportunities through formulation strategies, processing techniques, modification of protein properties, and emerging analytical methodologies that can support innovation are discussed. However, this review does not include consumer behavior, market aspects, and life cycle assessment of meat analogues. In summary, this review provides insights and future directions for optimizing extrusion-based PBMA production and advancing products with improved meat-like characteristics.

## 2. Plant Protein Ingredients for Extrusion

The selection of plant protein source plays a crucial role in the development of PBMAs, as protein composition, molecular structure, and techno-functional properties greatly influence the pasting, rheological, texture, nutritional value and sensory behavior of PBMAs [29]. This section explains the major plant protein sources and emerging plant protein sources used for the development of PBMAs.

### 2.1. Major Protein Sources

#### 2.1.1. Soy Protein

The most common plant protein source used in the production of PBMAs is soy protein. This dominance is primarily due to a well-established amino acid profile, favorable functional properties, and established global supply chains. In addition, its widespread application in commercial products such as tofu, soy milk, tempeh, and soy sauce has driven soybean cultivation and processing, ensuring availability and price stability. However, it is important to distinguish between native soy proteins within the seed matrix and commercial soy protein ingredients, as extraction, purification, and processing steps can alter protein structure, composition, and functionality. Commercial soy protein isolate is primarily composed of two globulin fractions: glycinin (11S globulin, representing 30–40% of total protein), with a total molecular weight of approximately 320–360 kDa, composed of acidic and basic polypeptide subunits linked by disulfide bonds, and β-conglycinin (7S globulin, 30–50% of total protein), with a total molecular weight of 150–200 kDa [29,35]. Glycinin and β-conglycinin contribute differently during high-moisture extrusion. Glycinin can promote structural integrity and gel strength by forming disulfide-linked networks through polymerization, whereas β-conglycinin enhances lipid emulsification and contributes to softer, more elastic gel textures [29,36].

Soy protein exhibits a relatively balanced amino acid composition but may lack sulfur amino acids (SAAs) such as methionine [16,37,38]. Despite SAA limitation, soy protein isolate has been reported to have a Digestible Indispensable Amino Acid Score (DIAAS) of 0.92 [39], indicating relatively high protein quality. In addition, soy protein possesses excellent functional properties and is widely available, making it suitable for the development of various soy-based products such as tofu, tempeh, and texturized soy protein [2,38,40].

#### 2.1.2. Pea Protein

The popularity of pea protein as an ingredient in PBMAs is due to its unique processing characteristics, including emulsification and foaming capacities that contribute to the formation and stabilization of protein networks, a high water-holding capacity that enhances moisture retention, and a relatively milder flavor profile [14,29,41,42]. However, pea protein structures are generally weaker than soy-based alternatives and often require hydrocolloids to improve texture and stability [17,43,44,45,46,47]. This is mainly attributed to the lower gelling capacity of pea protein and differences in protein composition, as soy globulins have higher solubility and gelation ability, promoting the formation of a stronger gel network [45,48]. Pea protein primarily consists of two major globulin fractions: legumin (11S), representing 8–21% of the total protein with a total molecular weight of approximately 360–400 kDa, and vicilin (7S), representing 21–55% of the total protein (18–41% cleaved forms + 3–14% uncleaved forms) with a total molecular weight of approximately 150 kDa. Altogether, these globulins account for about 50–60% of the total protein, with minor amounts of conviclin and albumins [49,50].

#### 2.1.3. Wheat Gluten

Wheat gluten stands apart from other plant proteins because of its viscoelastic properties, which arise from two main protein fractions: gliadin and glutenin, roughly 80–85% protein by dry weight [51,52]. Gliadins contribute viscosity and extensibility, while glutenins provide elasticity and strength [53,54]. These properties arise from the interaction of covalent and non-covalent bonds between the two proteins, which maintain the gluten network structure. Under extrusion-like conditions, glutens undergo relatively enhanced protein–protein and protein–water interactions, forming 3D gel structures via disulfide bonds, hydrogen bonds and hydrophobic interactions [54,55]. Wheat gluten can be widely used in PBMAs due to its ability to form fibrous protein films upon stretching, along with its functionality as a binding and structuring agent; however, it may pose potential risks such as gluten intolerance and allergies [56,57].

### 2.2. Emerging Protein Source: Faba and Lentil Proteins

A variety of alternative protein sources, including faba bean, lentil, mung bean, oilseed, algae, and insect proteins, have emerged as promising alternatives for the development of PBMAs [12,14,16]. Among these protein sources, faba bean protein (FBP) and lentil protein are discussed in detail below.

FBP is a promising plant protein source for the development of PBMAs due to its high protein content (25–35% dry basis) [58], cost-effectiveness, and functional properties, including emulsification, foaming, and gelation capabilities, which allow it to compete with conventional plant proteins. It is predominantly composed of globulins (69.5–78.1%), followed by glutelins (12.0–18.4%), prolamins (1.83–3.57%), and albumins (1.41–3.01%), with the relative distribution of these fractions varying according to cultivar and environmental conditions [59]. It primarily has 7s vicilin-type and 11S legumin-type proteins [29,58]. Recent studies have explored the application of fava bean protein in PBMA development, demonstrating its potential to form meat-like structures with improved texture and physicochemical properties [60,61,62]. However, further research is needed to optimize processing strategies and formulation approaches to improve the sensory attributes and consumer acceptance of faba bean-based PBMAs, particularly by addressing challenges associated with beany flavors and undesirable volatile compounds [58].

Lentil is an emerging plant-based protein source, containing approximately 24–30% protein, with its protein fraction consisting primarily of globulins (70%), followed by albumins (16%), glutelins (11%) and prolamins (3%) [63,64]. Its globulin fraction is primarily composed of vicilin-like (51%), legumin-like (35%), and 2S globulin (14%) proteins [65]. Legumin has a total molecular weight of approximately 320–380 kDa and is composed of polypeptide subunits linked by disulfide bonds, whereas vicilin has a molecular weight of 50–60 kDa and consists of glycosylated subunits [64]. However, variation in protein composition may occur among different lentil proteins and their processing conditions. It exhibits a favorable amino acid profile, providing adequate levels of most essential amino acids, although it is relatively limited in sulfur-containing amino acids (cysteine and methionine) and tryptophan [64]. Combining lentil protein with complementary cereal proteins can improve its overall amino acid balance. For instance, Boeck et al. [66] combined lentil protein with cereal proteins to develop plant-based milk alternatives and optimized the protein ratio to significantly improve the essential amino acid score. Therefore, combining lentil and cereal proteins represents a promising strategy for enhancing protein quality and improving techno-functional performance, which can be used in PBMA formulations.

## 3. Extrusion Technology: Mechanisms and Processing Parameters

Future plant-based meat analogue production needs better integration of multiple processing steps alongside a deeper understanding of how equipment design and processing technologies shape meat-like properties [7,21]. Although extrusion cooking remains the most scalable and industrially viable technology for PBMA production, achieving consistent meat-like structures remains challenging. This limitation arises from the complex interactions among raw material composition, protein functionality, processing parameters, and equipment design, where the combined effects on protein restructuring, fiber formation, and final texture are not yet fully understood. A proper understanding of these interactions is essential for optimizing extrusion conditions and producing texturized products with improved sensory and structural properties. Extrusion cooking represents a complex thermomechanical process in which plant proteins undergo controlled denaturation, molecular reorganization, and structural alignment to create fibrous, meat-like textures. There are two types of extrusion: low-moisture extrusion and high-moisture extrusion.

### 3.1. Low-Moisture Extrusion

The low-moisture extrusion (LME) process operates at relatively low moisture contents (typically below 35%), in which proteins are denatured and partially cross-linked with elevated temperature (120–180 °C) and shear, producing porous texturized vegetable protein (TVP) [14,67]. The final characteristics of the extrudates, such as density, expansion, texture, and porosity, depend on processing parameters such as moisture content, barrel temperature, pressure, screw speed, and die geometry [68]. LME can produce either dense-formed products or highly expanded porous, puffed, sponge-like and hard structures that often need rehydration before consumption [29,67,69,70]. In addition, LME possesses certain limitations in mimicking whole meat simulations. It reduces protein plasticity and molecular mobility, limiting structural alignment and producing less anisotropic textures, and is suitable for coarse products like ground meat analogues rather than whole-cut structures [21]. Therefore, it is mostly used in the food industry for burgers, patties, and other food preparations based on minced meat.

### 3.2. High-Moisture Extrusion (HME)

HME is a thermomechanical process that transforms protein through combined heat, shear and moisture (>40%), which involves mixing, cooking, kneading, and shaping, leading to changes in texture, size and flow properties of proteins [5,71,72,73,74,75,76]. During processing, proteins undergo denaturation, unfolding, and alignment, forming a viscoelastic protein melt. Molecular interactions, including hydrogen bonding, disulfide bonding, and hydrophobic interactions, contribute to the development and stabilization of structured protein networks. When the extrudates pass through a cooling die, the controlled temperature gradient and shear promote the alignment of protein molecules in the flow direction, improving texture consistency and mechanical integrity [21]. Unlike LME, HME operates at higher moisture levels (40–80%), enabling the formation of dense, fibrous, and anisotropic structures that closely resemble the texture and organization of animal meat [24,30,60,70,77]. Therefore, HME is considered one of the most promising technologies for producing next-generation plant-based meat analogues.

#### 3.2.1. Critical Processing Parameters During HME

Product quality in HME depends on various parameters, including temperature profile, moisture content, screw speed, barrel and screw configurations and die design. These parameters are closely interrelated with each other, which poses the core engineering challenge. The equipment parameters, ingredient properties and desired product characteristics must be optimized together, not in isolation [21], to obtain the desired characteristics in PBMAs. 

##### Moisture Content

The moisture content of a sample plays a key role in high-moisture extrusion by influencing protein–water interactions, phase separation, and the formation of the thermoplastic protein matrix. Higher moisture reduces material viscosity and extrusion resistance, resulting in lower friction, altered morphology, and larger pore structures in the final extrudates [77]. Water–protein interactions are critical for protein conformation, network formation, and texture, with water stabilizing the protein network and influencing gel firmness and compactness [78]. Water acts as a strong plasticizer in protein systems due to its small size, low glass transition temperature (Tg), and ability to form hydrogen bonds with protein molecules. Consequently, changes in moisture levels can significantly influence mechanical properties [79]. For instance, in a recent study by Elshamy et al. [61], the total target moisture content of faba, pea and soy proteins significantly correlated negatively with textural properties such as hardness, chewiness and cutting strength, while no significant effect on springiness was observed. Furthermore, this study highlighted inconsistencies in the literature regarding the optimal moisture content for texturization, with reported values ranging from 35% to 60%. Xia et al. [80] studied the fibrous structure of a meat analogue on yeast protein powder by varying the temperature (160–180 °C) and moisture content (55–65%). Their findings revealed that processing temperature impacted hardness, springiness, and chewiness differently depending on the moisture content. However, at a given processing temperature, increasing the moisture content generally reduced these textural properties in most samples. For instance, at 160 °C, hardness decreased from 122.31 N to 92.64 N, springiness decreased from 4.8 to 4.17 mm, and moisture content increased from 55% to 65%. Hu et al. [77] observed changes in mechanical properties and morphology under the effect of moisture content in a soy protein concentrate (SPC). The hardness of SPC extrudates decreased with an increase in the moisture content (50–70%), while morphological studies showed that increasing moisture produced larger, more elongated pores and enhanced fibrous structure at 58% and 62% moisture. However, excessive moisture (66–70%) reduced fiber clarity and resulted in irregular sheet-like structures. Furthermore, Hossain Brishti et al. [34] found that an optimal feed moisture of 49.3% enhanced the functional and rheological properties of texturized mung bean protein with a strong, stable gel network. This was attributed to greater disulfide bond formation compared with samples produced at 30% and 60% moisture levels. In contrast, lower (30%) and higher (60%) moisture conditions resulted in weaker gel structures due to excessive protein denaturation and weaker gel structures. A conceptual trend of how moisture content influences differences in HME parameters is presented in Figure 1. These studies indicate that while moisture is essential for fiber formation during HME, excessive moisture generally compromises mechanical strength, underscoring the need for optimized moisture levels to achieve desirable textural properties. Nevertheless, the reported optimum moisture contents vary among studies because moisture interacts with other processing variables, including barrel temperature, screw speed, cooling conditions, feedstock composition, screw configuration, and extruder design.

##### Temperature Profile

The extrusion system is composed of independently controlled barrel zones that allow feed to be directed towards sequential transformation. During the development of PBMAs, multi-zone temperature profiles are used, which generally rise from 20 to 25 °C in the feed zone and reach 140–160 °C in the melting zone, exceeding the denaturation temperature of most plant proteins [23,74]. The primary reason for the melting zone is to promote denaturation of proteins, maintain enough viscosity for flow, die filling and inactivate heat-labile components.

Barrel temperature plays a vital role in structuring high-moisture meat analogues. As illustrated conceptually in Figure 2, changes in barrel temperature can influence key HME processing parameters, which in turn affect the resulting product structure and quality. Accordingly, the effect of barrel temperature on product structure has been reported in several studies. For example, Chew et al. [81] compared the mechanical and macrostructure characteristics of different soy concentrate extrudates with a 10-zone temperature profile. Zones 8 to 10 were examined at various combinations of extrusion temperature, i.e., 90, 110 and 130 °C. Their study showed that as the processing temperature rose from 110 °C, the extrudates developed fibrous macrostructures for Solcon S, Wilcon, and YX702 soy concentrates, whereas ALPHA^®^ 8 IP, a functional soy protein concentrate manufactured by Solae, exhibited fibrous macrostructures after 130 °C. This difference may be attributed to variations in the rheological response and structural resistance of soy concentrates during extrusion. ALPHA^®^ 8 exhibited greater resistance to heat and shear-induced structural breakdown, suggesting that higher thermal energy was required to facilitate protein unfolding, liquefaction, and subsequent alignment into anisotropic fibrous structures. Furthermore, with increasing temperature, the hardness and chewiness of the extrudates increased, likely attributed to a greater degree of protein unfolding, aggregation and more intermolecular interactions. In another study, different plant proteins at different moisture contents were optimized for the development of a meat analogue, and it was found that a fiber-like structure developed in extruded products above 110 °C [82]. Recently, Toldrà et al. [60] examined how high-moisture extrusion conditions affect the physicochemical and techno-functional properties of faba bean protein concentrates; they found that fibrous HME products can be produced using 50–52% solids at 143–150 °C. Moreover, Xia et al. [80] reported that the fibrous structure and strength of yeast protein powder meat analogue improved by increasing the processing temperature from 160 to 180 °C at constant moisture, with 180 °C producing highly developed fibrous structures (fibrous degree > 1.60). This was associated with intensified phase separation at higher temperatures, which promoted protein alignment and the formation of a stronger fibrous network during HME. However, higher temperatures can cause excessive aggregation, which negatively affects the sensory attributes of a meat analogue.

Overall, extrusion temperature plays a critical role in modifying protein physicochemical and structural characteristics, thereby promoting meat-like fiber formation through protein denaturation, unfolding, aggregation, and alignment. Increasing temperatures provide sufficient thermal energy to disrupt native protein structures and enhance intermolecular interactions, facilitating the development of a fibrous network. However, the optimum temperature can vary among protein sources due to differences in protein structure, aggregation behavior, and resistance to heat- and shear-induced breakdown. Furthermore, direct comparison of temperature effects among studies remains challenging because the structural changes induced by temperature are strongly influenced by interacting processing factors, including moisture content, screw speed, cooling conditions, barrel length, and extruder design. Therefore, it can be hypothesized that an optimal temperature range is necessary to achieve controlled protein restructuring, as insufficient or excessive temperatures may limit desirable fiber development.

##### Screw Speed

Screw speed (SS) is a key extrusion parameter that controls shear rate and material residence time, with higher screw speed promoting protein deformation, melt development, die pressure, and fiber formation [83]. Sun et al. [84] investigated the effect of different screw speeds (100–160 rpm) on the quality attributes of soy protein isolate (SPI) extrudates. Their study revealed that increasing screw speed enhanced the shear rate, with approximately a 60% increase observed at 160 rpm. Furthermore, the hardness of the extrudates increased when screw speed was increased from 100 to 140 rpm due to improved protein melting and structure formation; however, hardness decreased at 160 rpm because the reduced residence time limited the development of a well-formed fibrous structure. At lower screw speeds, SPI formed more aggregates with darker color and rougher surfaces, which was attributed to the longer residence time that promoted excessive protein aggregation. The impact of increasing screw speed on different HME parameters is shown in Figure 3.

In another study, Rajendra et al. [85] examined the effect of extrusion parameters on hempseed protein and found that increasing screw speed to 400 rpm provided a higher texturization index, suggesting that protein degradation was limited under the investigated conditions, with a maximum barrel temperature of 120 °C. However, Maung et al. [86] reported contrasting results, where increasing screw speed increased specific mechanical energy (SME), resulting in a reduction in the degree of texturization. For instance, at 55% feed moisture, increasing screw speed from 150 to 200 rpm decreased the degree of texturization by approximately 27%. The increase in SME was attributed to greater shear and frictional forces within the extruder, with the highest SME observed under low feed moisture, high barrel temperature, and high screw speed conditions. Similar results were reported by Ribeiro et al. [87], who found that increasing screw speed from 300 to 400 rpm resulted in reduced hardness of the meat analogues produced. These differences indicate that higher shear forces generated during higher screw speeds enhanced protein denaturation and molecular rearrangement, reducing melt viscosity and melt pressure at the die exit and producing softer-textured meat analogues under higher SS conditions.

These studies collectively suggest that increasing screw speed to an optimum level enhances mixing, shear, and friction, which promotes protein unfolding, alignment, and fibrous structure development. However, beyond a critical screw speed, excessive shear and increased SME can lead to excessive protein denaturation and disruption of intermolecular interactions, while reduced residence time limits the alignment and cross-linking of proteins required for the formation of an organized fibrous network. Therefore, careful optimization of screw speed is required to achieve the desired degree of texturization, as the relationship between screw speed and product quality is influenced by the balance between shear intensity, residence time, and mechanical energy input.

##### Screw Configuration

Screw configuration influences material transformation, mixing efficiency, fill level, and energy input during extrusion. Twin-screw extruders are classified into four main types based on screw arrangement and rotational direction: co-rotating non-intermeshing, co-rotating intermeshing, counter-rotating non-intermeshing, and counter-rotating intermeshing systems. Among these, co-rotating intermeshing twin-screw extruders are commonly used for high-moisture extrusion (HME) due to their efficient mixing, conveying ability, and shear distribution, which promote fibrous structure development [88]. Limited studies are available that show the effect of screw configuration on meat analogues. A brief summary of the impact of screw configuration on HME parameters is shown in Table 1. Zhang et al. [89] reported that flow-restricting elements such as reversed helix screws, staggered elements, and narrow kneading discs increase the screw fill degree, thereby increasing mechanical energy input. For instance, these elements increased the screw fill degree by up to 379%, resulting in an increase in SME of approximately 154%. These observations suggest that flow-restricting elements impede forward material transport, increasing barrel filling and localized pressure, which enhances shear stresses and promotes the protein unfolding and alignment required for fibrous structure formation. Furthermore, moving the reverse kneading element closer to the die (from 150 to 50 mm) increased SME by up to 131%, possibly because the material experiences higher viscosity and flow resistance near the die.

Similarly, Li et al. [90] reported that the incorporation of reversing elements and kneading blocks improved extrudate hardness, chewiness, and fibrous structure (from 1.01 to 1.27), with post-positioned reversing elements showing enhanced fiber formation. This is likely because the downstream reversing element increased melt reflux, residence time, and shear, promoting protein unfolding and the development of a more organized anisotropic fibrous structure. However, excessive kneading may not always be advantageous, as reducing the number of kneading discs lowered shear and mechanical energy input while improving structural integrity and reducing oil leakage in oil-containing formulations [91]. These findings indicate that the selection and arrangement of screw elements play a critical role in regulating energy transfer, protein restructuring, and the development of desirable fibrous structures during high-moisture extrusion.

Overall, restrictive screw elements, particularly reverse and post-positioned kneading elements, tend to increase material fill, SME, and shear, thereby promoting fibrous structure formation and, in some cases, greater hardness and chewiness. However, these effects are highly dependent on element geometry and position, as well as formulation, moisture content, screw speed, temperature, and cooling-die conditions.

##### Cooling Die

The cooling die in twin-screw extrusion is critical for developing fibrous and anisotropic structures by controlling temperature, morphology, and directional alignment of extrudates [72,92,93]. In the cooling die, bio-thermo-fluid-dynamic effects regulate cooling-induced flow behavior and matrix properties to promote multilayered fibrous structures in high-moisture meat analogues [94]. Choi et al. [92] investigated the effect of cooling die temperature on the texture of plant-based meat analogues and reported that increasing the cooling die temperature reduced the hardness of high-moisture meat analogues by approximately 17.5% while improving protein alignment and texturization by 6.7%. Similarly, Gräfenhahn and Beyrer [95] evaluated the effects of cooling temperature and shear rate on the textural development of pea protein-based meat analogues using a rotating cooling die system. They found that increasing the cooling die temperature to 80 °C enhanced hardness, anisotropy, and fibrous structure formation, mainly through disulfide bond-mediated protein aggregation, whereas higher cooling shear rates had limited effects on hardness and negatively affected anisotropy. In another study, Zheng et al. [96] investigated the influence of dynamic elongation flow generated by a multihole die under different cooling temperatures on the structure and texture of soy protein extrudates, observing that optimal cooling temperatures (30–40 °C) promoted anisotropic, fiber-rich structures, with pronounced fiber alignment observed at 40 °C. This improvement was attributed to multihole die-induced elongational flow, which generated separated and oriented fiber structures within the protein melt. At 40 °C, the increased rate of heat transfer from the surface to the center of the extrudate enabled rapid stabilization of these elongated fibers, preventing their reaggregation and allowing them to interconnect into an anisotropic structure. In contrast, excessively high cooling temperatures delayed solidification, causing fiber reaggregation, whereas lower temperatures (<30 °C) altered protein rheological behavior and increased molecular aggregation, resulting in reduced fiber orientation. A brief summary of the effect of the cooling die on different HME parameters is presented in Table 2.

Beyond cooling temperature, die geometry is another important factor influencing extrusion performance, protein restructuring, and textural development. Zhou et al. [93] investigated the effects of die geometries (circular, square, and flat) on pea protein isolate (PPI) extrudates and found that a flat die reduced specific mechanical energy (669.99 kJ·kg^−1^) by increasing die pressure (4.34 MPa) and mass flow rate (299.40 g·min^−1^) while promoting densely aligned fibrous structures with improved mechanical strength. The enhanced fibrous structure in flat die extrudates may be attributed to the flatter geometry and larger outlet area, which promote rapid heat transfer, improved protein hydration, and the formation of a denser protein network with a higher proportion of bound water. Furthermore, die geometry also affected moisture distribution, particularly the fractions of bound and non-flowing water. Similarly, Guyony et al. [97] demonstrated that die dimensions, particularly die height and die length, are critical determinants of fiber formation during high-moisture extrusion of soy protein concentrates. Compared with a 2 mm die, a 5 mm die promoted the development of temperature gradients and velocity profiles, facilitating protein alignment and anisotropic structure formation. In contrast, the 2 mm die resulted in more homogeneous cooling and plug flow, limiting fiber development. Furthermore, increasing die length from 150 to 600 mm progressively improved fiber orientation by extending the cooling and alignment stages within the die. Thus, the combined influence of cooling strategy, die shape and dimension suggests that extrusion-induced structural development depends on the balance between flow-induced alignment, protein aggregation, and moisture redistribution, which leads to the final fibrous anisotropy of PBMAs. Different studies (Table 2) show that an optimum cooling die and temperature configuration is required to obtain desirable characteristics in PBMAs.

Overall, the critical processing parameters in HME, including moisture content, barrel temperature, screw speed, screw configuration and cooling die conditions, play a vital role in determining the final structural and textural characteristics of PBMAs. Their combined optimization is necessary to achieve desirable product quality, as these parameters are highly interdependent. A brief summary of previous studies on the extrusion of PBMAs is presented in Table 3.

### 3.3. General Molecular Transformations During Extrusion

HME has scaled up commercially; however, the molecular-level changes that occur during processing still remain incompletely characterized due to the complex and dynamic interactions among heat, shear, moisture and protein, which are difficult to monitor in real time. Vargas et al. [104] identify this as the most significant knowledge gap, as plant protein alterations during HME and their effect on digestibility and nutritional value are largely understudied. The general molecular transformations induced during HME at different zones are presented in Figure 4 and discussed in the following sections.

#### 3.3.1. Denaturation and Unfolding

Thermal, chemical, and mechanical treatments can alter protein molecular structures and consequently influence their functional properties. Extrusion processing achieves these structural modifications through the combined effects of heat, shear, and pressure within a continuous flow system [105,106]. These combined effects disrupt weak non-covalent interactions, including hydrogen bonds, electrostatic interactions and hydrophobic associations, causing denaturation and unfolding of protein structures. The melting zone of the extruder, often reaching temperatures between 140 and 160 °C, promotes protein denaturation [23,74]. Furthermore, high screw speeds and specific screw configurations, such as the use of flow-restricting reversed helix screws or kneading discs, intensify shear stress and mechanical energy transfer [84,86,89], with the extent of denaturation depending on cumulative energy input, residence time, protein source, and moisture content. As plant proteins naturally exist in globular, multimeric forms, processing is required to transform them into anisotropic fibrous structures that mimic the texture of meat [18,27]. During this transformation, protein denaturation plays a critical role by exposing reactive functional groups that promote intermolecular bonding and protein aggregation, ultimately facilitating fiber formation in plant-based meat analogues (PBMAs) [107].

#### 3.3.2. Protein Alignment and Network Formation

During extrusion, proteins undergo denaturation and liquefaction, enabling molecular mobility and flow-induced restructuring. A high moisture content acts as a plasticizer, facilitating protein rearrangement, while elevated extrusion temperatures (120–150 °C) weaken non-covalent intermolecular interactions, allowing the protein melt to deform under shear [107]. Under shear flow, proteins align in the direction of flow, promoting partial unfolding and β-sheet formation, which facilitate the formation of protein aggregates stabilized by hydrogen bonding, disulfide bonds and hydrophobic interactions. These aggregates serve as structural precursors for fibrous formation in plant-based meat analogues (PBMAs) [29,107].

As the aligned protein melt enters the cooling die, the temperature gradient promotes gradual solidification, while non-covalent interactions strengthen and disulfide bonds stabilize, increasing viscosity and fixing the aligned protein network. Cooling also suppresses expansion at the die exit, resulting in a stable cross-linked gel structure that preserves the anisotropic fibrous morphology of PBMAs [21,108]. Thus, shear-induced alignment generates structural precursors, whereas cooling stabilizes and preserves the final fibrous architecture.

## 4. Current Challenges in Extrusion-Based Meat Analogues

Despite growing consumer interest in plant-based meat alternatives, they continue to represent only a minor segment of the broader meat market [28]. This is because they still fail to match the fibrous texture, juiciness, appearance and sensory experience of animal meat at a price consumers find acceptable [10,20,21,29]. Therefore, enhancing the textural and sensory characteristics of PBMAs is one of the most significant challenges facing the industry. Achieving this characteristic requires a deeper understanding of protein source, extraction, and composition, as well as the complex interactions between ingredient functionality and extrusion processing conditions that govern the formation of meat-like structures.

### 4.1. Textural and Structural Limitations

Consumer acceptance of plant-based meat analogues (PBMAs) largely depends on their ability to replicate the hierarchical structure and texture of animal meat, particularly its characteristic fibrous architecture [4,70,109]. Unlike animal muscle, which is organized across multiple hierarchical scales through muscle fibers and connective tissue, PBMAs develop meat-like fibrous characteristics primarily through processing-induced protein restructuring, including denaturation, gelation, and fiber formation [19,20,55]. Consequently, reproducing the complex fibrous and textural structure of meat remains one of the greatest technical challenges in PBMA development, driving the need for advanced structuring technologies [5,13,18].

The limited fibrous structure of current PBMAs results in inferior textural properties, including lower tensile strength, reduced springiness, and suboptimal hardness compared with conventional meat. The texture felt during mastication and across cooking methods remains challenging, largely because PBMAs lack intermuscular fat, which contributes to tenderness, juiciness, lubrication, structural integrity, flavor release, and desirable cooking behavior through its unique melting and thermal properties [2,30,110].

Beyond textural characteristics, differences in composition also influence cooking performance. Although PBMAs generally exhibit higher water-holding capacity and reduced shrinkage than beef, resulting in improved cooking yield, they typically develop a softer and less cohesive texture after cooking [111]. Similarly, texture profile analysis has shown that plant-based burgers possess lower hardness, gumminess, and chewiness than beef burgers, largely due to higher water retention and differences in the composition and crosslinking of their biopolymer networks [22]. These findings highlight that mimicking cooking yield alone is insufficient to reproduce the mechanical and sensory characteristics of conventional meat. To address these limitations, various formulation strategies, including the incorporation of hydrocolloids, fats, and polysaccharides, have been explored. However, the use of certain hydrocolloids such as methylcellulose may conflict with clean-label expectations or introduce potential allergen concerns [29,112]. Therefore, mimicking textural properties requires balancing functional improvements with consumer expectations for simple and sustainable formulations.

Overall, the inability of current PBMAs to replicate the hierarchical organization of animal muscle remains a fundamental barrier to achieving real meat-like quality. Structural deficiencies across multiple length scales not only compromise texture and mechanical performance but also limit the effective integration of water and fat within the matrix. Consequently, future advances are likely to depend on multiscale structuring strategies capable of simultaneously recreating the fibrous, aqueous, and lipid architectures that underpin the sensory attributes of conventional meat.

### 4.2. Sensory Challenges

The sensory characteristics of meat are difficult to replicate and remain one of the most significant challenges in the development of PBMAs. Beyond texture, consumer acceptance is strongly influenced by flavor, aroma, visual appearance, and overall mouthfeel [5,16,28,30], all of which arise from complex interactions between proteins, lipids, pigments, and thermal reactions during cooking. Because plant-derived ingredients differ fundamentally from animal tissues in their chemical composition, recreating these sensory attributes requires extensive formulation and processing interventions.

The primary sensory challenge is the presence of inherent off-flavors in plant proteins and meaty aroma replication. Legume-based products often exhibit undesirable sensory attributes, including beany, grassy, green, rice bran-like, and sour notes, resulting mostly from hexanal and 2-pentylfuran [113,114,115]. Although ingredient refinement and breeding strategies have reduced the intensity of these undesirable flavors, they remain a major barrier to consumer acceptance. Processing can further complicate flavor development. High-temperature extrusion promotes chemical reactions among protein-rich ingredients, such as Maillard reactions and oxidation, which are the main contributors to color, texture and flavor in PBMAs [7,20,115]. However, excessive processing can potentially produce off-flavor compounds through enzymatic oxidation [30,116]. Consequently, many PBMAs rely on complex flavor systems containing higher-flavor ingredients/additives to achieve a complex aroma of real meat while masking the undesirable native flavors and aftertaste of plant-based raw materials [20,21,112,117]. Additionally, off-flavors can be reduced by masking undesirable flavors, preventing their formation, or removing them from raw materials through strategies such as spice and polysaccharide addition, fermentation, and germination [116].

In addition to flavor, visual appearance plays a critical role in shaping consumer perceptions of product quality. A particularly difficult challenge is replicating the characteristic color transition of red meat from red in the raw state to brown during cooking. This is due to the absence of myoglobin and hemoglobin in plant ingredients. Recently, Qian et al. [115] investigated the effect of color by incorporating Pichia-derived porcine myoglobin (PMb) and bovine hemoglobin (BHb) into high-moisture extruded soy–wheat gluten meat analogues. The addition of PMb significantly enhanced product redness and darkened the extrudates, with redness (a*) increasing from 5.51 to 6.44 at 0.5% PMb and reaching 8.33 at 1% PMb, indicating improved meat-like color development. However, its adoption is constrained by regulatory approvals, as it is not yet approved as a food ingredient.

Various colorants such as annatto, turmeric, carotene, cumin, leghemoglobin, beet juice, beet powder and carrot extracts are commonly used to replicate the presence of myoglobin [3,30,118]. However, the application of natural colorants has technological limitations, such as lower color intensity and stability compared to synthetic dyes. Their performance is highly sensitive to processing conditions such as pH, heat, moisture, and screw speed, making it essential to understand their behavior during thermomechanical processing in extruded meat analogues [31].

Overall, the sensory gap between PBMAs and conventional meat arises from fundamental differences in the chemical composition of plant and animal proteins. Inherent off-flavors, difficulties in replicating meat-like color transitions, and limitations in aroma precursor availability collectively hinder the development of authentic sensory experiences similar to those of real meat. Therefore, future strategies should integrate proper ingredient selection, flavor engineering, color optimization, and process control to more closely reproduce the dynamic sensory changes that occur during meat cooking and consumption.

### 4.3. Nutritional Limitations

Despite significant advances in PBMA processing, it still faces important nutritional challenges that limit its ability to replicate the nutritional profile of animal meat. These limitations are primarily related to protein quality, micronutrient availability, and nutrient digestibility and bioavailability. Plant proteins contain one or more limiting essential amino acids. Cereal proteins are often lysine-deficient; for instance, wheat gluten provides approximately 0.3–1.1 g lysine per 100 g protein, whereas animal meat can provide up to 9.0 g per 100 g of total amino acid (TAA) [119,120,121]. Similarly, legume proteins are low in methionine and cysteine—soy, pea, and faba bean provide 0.87–1.29 g methionine and 0.85–1.55 g cysteine per 100 g protein, compared with about 3.0 g methionine and 1.7 g cysteine per 100 g TAA in beef meat [59,121]. These differences in amino acid composition are also reflected in DIAAS values, with reported scores of 0.92 for soy protein isolate, 0.66 for pea protein isolate, and 0.39 for wheat protein, compared with 1.08 for milk protein and 0.90 for whey protein [39]. However, DIAAS values may differ for a given protein source depending on processing conditions, formulations and assessment methods. Extrusion may further reduce lysine availability through Maillard reactions, with retention reaching 51% to 89% under harsh processing conditions [122,123].

Furthermore, increasing consumption of plant-based products may increase the risk of inadequate intake of nutrients commonly found in animal-derived foods, such as vitamin B12, vitamin D, iron, and zinc [20,117,124,125]. Therefore, fortification is often necessary to compensate for nutritional gaps and improve the nutritional profiles of plant-based products. In addition, extrusion processing can reduce vitamin stability and retention, with only 44–62% of B vitamins retained [29,126], further highlighting the need for appropriate nutrient supplementation. Moreover, extrusion can also improve plant protein digestibility by promoting protein denaturation and hydrolysis and reducing antinutritional factors, thereby enhancing the release of amino acids and peptides. However, excessive heat may cause losses of heat-sensitive amino acids and reduce protein quality through oxidation, aggregation, and Maillard reactions [104].

### 4.4. Processing-Related Issues

Extrusion heating can improve protein availability, digestibility, and microbial safety and reduce antinutritional factors by unfolding macromolecules and inactivating harmful compounds. However, intense thermomechanical conditions may produce heat-induced compounds. This can promote protein cross-linking, Maillard reaction products, advanced glycation end products (AGEs), starch–protein interactions, and aggregation, potentially reducing digestibility and reactive lysine content and altering allergenicity and toxicological properties [127]. Moreover, soy protein isolates containing high levels of asparagine (9.0–9.4 g/100 g) [128] may react with reducing sugars above 120 °C and form acrylamide [129]. In addition, thermal degradation can generate furans, whereas lipid oxidation produces aldehydes and ketones, which can affect product quality and safety.

PBMA production generally has lower environmental impacts than animal meat; however, the extent of these benefits depends largely on ingredient selection and processing requirements. While alternative protein sources are often promoted for their potential environmental and health benefits, their adoption may also have indirect impacts, and highly processed plant-based alternatives may still contribute to environmental burdens due to processing-related emissions and resource use [130,131]. Accordingly, more comprehensive life cycle assessments that incorporate biodiversity, soil health, eutrophication and acidification are required to accurately determine the environmental effects of PBMAs [132]. Similar to this, the findings of Detzel et al. [131] suggest that extruded plant-based meat substitutes offer environmental advantages over chicken meat across most impact categories; however, these advantages do not extend to cumulative energy demand, process water demand, and land use.

Most PBMAs use multiple ingredients such as hydrocolloids, starches, flavors, colorants, spices, etc., each serving as a functional ingredient but collectively undermining the minimally processed image many consumers expect from PBMAs. Furthermore, PBMAs are found to be costly compared to conventional meat. For instance, Lou et al. [133] found that the median price of PBMAs in Malaysia was below Malaysian Ringgit (MYR) 7 (USD 1.6) per 100 g, compared with MYR 1.14 (USD 0.3) per 100 g for poultry and MYR 3 (USD 0.7) per 100 g for chunks/fillets/strips, making PBMAs substantially expensive and deterring 44% of consumers from purchasing these products. A similar premium price has been reported for new food innovations, with plant-based products costing up to 67% more than conventional meat [134]. This price premium is largely attributed to the processing and formulation complexity of plant-based products, which require additional ingredients such as flavoring agents, structured fats, and texturizing components to replicate the flavor, texture, and functionality inherently present in animal meat.

## 5. Opportunities and Emerging Solutions

### 5.1. Advanced Formulation Strategies

Recent research has increasingly focused on advanced formulation strategies to overcome challenges related to the texture, flavor, and nutritional composition of PBMAs. Among the strategies, structuring systems for PBMAs based on hydrogels and lipids have rapidly increased due to their ability to mimic the key characteristics of animal meat. Hydrogels derived from natural polymers, such as proteins and polysaccharides, are widely used in the food industry due to their availability, low toxicity, biocompatibility, and biodegradability. With appropriate design, these hydrogels can also achieve desirable swelling behavior and mechanical properties that can be used to improve the texture, water-holding capacity, and fat replication of PBMAs [23,24,135]. Plant proteins tend to show strong gelation and hydration properties, while polysaccharides such as alginate, pectin, and cellulose contribute to thickening, emulsification and structural reinforcement. However, plant protein-based hydrogels often provide weak mechanical strength, requiring modification of the network structure and functional properties of the protein [55].

Although hydrogels play a critical role in replicating the structural properties of meat, they alone cannot fully reproduce the juiciness, tenderness, and flavor release associated with animal fat. The incorporation of lipid systems is one of the most effective strategies for improving the juiciness, flavor release, and tenderness of plant-based meat analogues (PBMAs) [12,15]. Various lipids, including stearic acid, soybean, coconut, peanut, rapeseed, palm, and sunflower oils, are used to enhance sensory quality and nutritional value; however, inappropriate selection of oil type, oil level, or protein source can result in poor product structure and undesirable flavor [15,20,136]. Moreover, Ali et al. [110] highlighted the limitations of coconut and palm oils, including their unsuitable melting behavior, high saturated fat content, sustainability concerns, and poor oil stability, and proposed structured lipid systems, such as oleogels, emulsion gels, Pickering emulsions, and bigels, as promising alternatives to better replicate animal fat while improving sensory quality, nutritional value, and sustainability.

Heme proteins have emerged as promising ingredients for improving the sensory properties and nutritional profile of PBMAs. For instance, Meng et al. [137] reported that a control formulation without added hemoglobin protein exhibited a yellowish color characteristic of soy protein, whereas formulations supplemented with 1.0% leghemoglobin or 1.0% myoglobin developed a pink, meat-like appearance. These findings suggest that the incorporation of heme proteins can effectively enhance the visual appeal of PBMAs by imparting a color that more closely mimics fresh meat. Furthermore, Impossible burgers use soy leghemoglobin, a soy-derived heme protein with structural and functional similarities to hemoglobin and myoglobin, to impart a meat-like color, thereby enhancing the visual appeal of the plant-based meat analogue [138].

### 5.2. Hybrid and Integrated Processing Technologies

The production of meat analogues is moving beyond conventional extrusion toward integrated digital and hybrid structuring technologies, including 3D food printing, artificial intelligence (AI) and machine learning (ML) for customizable and sustainable production [3,26,139]. Extrusion alone cannot replicate a muscle’s multiscale hierarchical architecture. Therefore, recent reviews suggest that combining the working principles of multiple technologies integrated with extrusion may be a promising approach to improving the structure and texture of PBMAs [25]. 3D printing can enable alternating layers of different protein compositions, incorporation of fat layers through multi-material printing, and custom geometries for specific product types. It can be used in the production of a wide range of complex and customized products, including meat analogues, with precise control over texture and flavor. Recent advances in multi-material and multi-nozzle printing, together with complementary technologies such as electrospinning and encapsulation, can further improve the structural design and controlled flavor release in alternative protein products [3,140].

In a recent study by Ghimire et al. [26], a 3D-printed meat analogue from fermented finger millet showed significant improvement in rheology, particularly an increase in hardness, springiness, gumminess, and chewiness, because of the increased interaction between protein and starch during fermentation. Similarly, Liu et al. [141] developed a meat analogue using mycelium-enhanced plant protein–polysaccharide hydrocolloids (containing k-carrageenan and locust bean gum) with enhanced structural stability, mimicking the muscle fiber profile of chicken breast and salmon. Other technologies, such as precision fermentation, can be integrated with existing technologies to mimic real meat. Precision fermentation can offer opportunities for PBMAs by enabling the production of specific proteins and functional ingredients that are difficult to obtain through conventional plant-based approaches. These include animal-identical proteins, heme proteins (leghemoglobin, myoglobin), and targeted flavor precursors [142], which can contribute to meat-like color and flavor. The incorporation of these fermentation-derived ingredients into HME formulations may further enhance the sensory, nutritional and functional properties of meat analogues.

### 5.3. Novel Protein Sources and Ingredient Innovation

A successful future for meat analogue development will also depend on the identification of novel protein sources beyond soy, pea, and wheat gluten. This can help reduce supply chain concentration and provide opportunities for functional differentiation and nutritional improvement. Other sources, such as faba bean, lentil, chickpea, mung bean, and oil seed proteins, are the most viable options as alternatives for use in the production of meat analogues [14,16,29] but may require further processing for removing antinutritional factors, color improvement, and flavor correction before extrusion. Furthermore, plant protein concentrates can also emerge as promising alternatives to protein isolates for PBMA production. Dry-fractionated pea protein concentrates can provide comparable fibrous structure formation while reducing the need for energy- and water-intensive isolation processes, whereas hemp protein concentrate has demonstrated potential to replace up to 60% of soy protein isolate while maintaining comparable textural properties [42,143].

Beyond novel protein sources, pre-extrusion protein modification strategies, including thermal, pressure, ultrasound, pH-shifting, enzymatic, microwave, and fermentation treatments, can improve functionality, reduce antinutritional factors, and enhance flavor. For example, Eslami et al. [46] demonstrated that enzymatic cross-linking of pea protein isolate (PPI) and low concentrations of collagen using transglutaminase (TGase), particularly in combination with NaCl, significantly enhanced gel strength and promoted the formation of stronger protein networks through intermolecular cross-linking. However, excessive TGase concentrations induced phase separation and reduced gel strength, highlighting the importance of optimizing enzymatic treatment conditions to achieve the desired functionality prior to further processing. Such improvements in protein network formation may provide a favorable starting material for HME by enhancing the structural integrity of protein blends. Furthermore, Zhang et al. [144] studied the application of TGase to improve the fibrous structure of different extrudates. Their study concluded that textural properties of high-moisture extrudates were strongly influenced by both protein type and TGase treatment. TGase improved fibrous structure and springiness in some proteins (especially SPI and PPI) but softened peanut protein powder (PPP) extrudates, reducing hardness, shear force, and tensile resistance while increasing hardness in SPC. This means that TGase-induced restructuring depended on protein composition and extrusion conditions. Furthermore, a recent study reported that pH significantly affects protein modification during high-moisture extrusion by influencing protein interactions, viscosity, and disulfide bond formation. Neutral pH promoted greater protein unfolding and cross-linking, producing fibrous, anisotropic structures, whereas acidic pH resulted in more isotropic textures. The authors also found that higher dietary fiber levels reduced protein network formation, demonstrating that both pH and the protein-to-fiber ratio are key factors governing the structure and texture of meat analogues [145].

### 5.4. Advanced Analytical and Characterization Approaches

Peptidomics is a mass spectrometry-based peptide analysis that can characterize Maillard products, oxidized amino acids, and cross-linked peptides formed during extrusion; assess protein digestibility through digestion studies; identify bioactive peptides; and detect protein degradation or quality issues [104]. However, its application to PBMAs is still limited by challenges in sample preparation, data acquisition, and data interpretation related to complex food matrices. Wang et al. [146] studied the peptidomic potential of PBMAs as sources of bioactive peptides. Their findings revealed that although PBMA proteins exhibited lower digestibility than beef proteins, their digestion products showed a comparable amino acid profile, highlighting the potential of PBMAs as precursors of bioactive peptides. Therefore, integrating peptidomics studies into PBMAs could provide molecular-level findings linking protein structure, digestibility and nutritional function, helping to address the knowledge gap in the field.

Understanding process–structure–function relationships in high-moisture extrusion remains challenging due to the complex interactions between heat, flow, phase separation, and protein restructuring. Recent advances in modeling and dead stop experiments in extrusion have provided insights into anisotropic structure formation; however, further research is needed to optimize processing conditions and develop PBMAs with improved texture, nutrition, and functionality [10,82,94]. Furthermore, AI-driven predictive modeling can help elucidate glycation–structure–function relationships in plant proteins [147], which can provide insights into how glycation-induced molecular changes influence protein functionality, texture development, and nutritional properties in PBMAs. As sufficiently large datasets become available, the integration of deep learning, multi-source data analysis, and inverse design approaches offers a promising strategy to predict and optimize glycation patterns, facilitating the development of PBMAs with enhanced meat-like texture, sensory attributes, and functional performance. One of the recent studies by Gulzar et al. [139] explored the potential of AI-based modeling for optimizing soy protein concentrate high-moisture meat analogues (SPC-HMAs) designed to mimic chicken meat by incorporating an optimal combination of sorbitol and moisture. Their findings revealed that genetic algorithm (GA)-based optimization was able to predict and replicate texture profiles comparable to real chicken meat. However, optimal processing conditions for developing beef analogues could not be accurately determined due to model limitations, suggesting reconsideration of the experimental range and emphasizing the need for more comprehensive datasets and refined AI models for precise prediction and optimization of diverse meat analogue systems. AI-driven modeling in food processing is constrained by limited datasets, risks of overfitting, difficulties in data acquisition, and limited model interpretability, which restrict model robustness and generalizability [148]. Therefore, future AI-based modeling should prioritize the development of large, diverse, and high-quality datasets, together with experimental validation, before predictive models can be generalized across formulation and processing conditions. Future studies should also expand beyond chicken and beef to develop high-moisture extruded analogues of lamb, pork, and seafood, expanding the diversity of PBMAs.

## 6. Conclusions

High-moisture extrusion remains the most established and scalable technology for producing fibrous, meat-like structures from plant proteins, making it central to the development of PBMAs. Despite considerable progress, current PBMAs continue to face challenges in replicating the texture, sensory attributes, nutritional quality, and affordability of conventional meat. These limitations largely arise from the intrinsic properties of plant proteins, which do not naturally assemble into the hierarchical structures found in various animal meats, as well as the complex interactions among HME processing parameters that govern protein transformation, fibrous structure formation, and, ultimately, textural properties. Therefore, a deeper mechanistic understanding of the process–structure–function relationship during HME is essential to establish how extrusion parameters influence protein functionality and structure development and to design high-quality PBMAs.

To overcome these challenges, a multidisciplinary approach that combines advances in raw material selection, protein blending and modification (e.g., through pH adjustment, salt treatment, and fiber incorporation) and innovative formulation approaches is required. Emerging analytical techniques, such as peptidomics, together with artificial intelligence-driven modeling, can offer promising opportunities to improve our mechanistic understanding and optimize the HME process. In this context, this review provides several technical implications and pathways for the food industry. For instance, achieving realistic fat replication in PBMAs cannot rely solely on simple oil addition; instead, the integration of structured lipid systems, such as oleogels, emulsion gels, bigels, and Pickering emulsions, is essential to replicate the physicochemical and sensory characteristics of real meat. Future research should therefore focus on the broader requirements for successful PBMA development, including consumer acceptance, nutritional adequacy, economic feasibility, and environmental sustainability. By integrating innovations in ingredient functionality, processing technologies, and analytical tools, the next generation of extrusion-based PBMAs will be well positioned to deliver products that more closely resemble conventional meat in structure, sensory quality, and nutritional performance while supporting the transition toward more sustainable food systems.

## Figures and Tables

**Figure 1:**
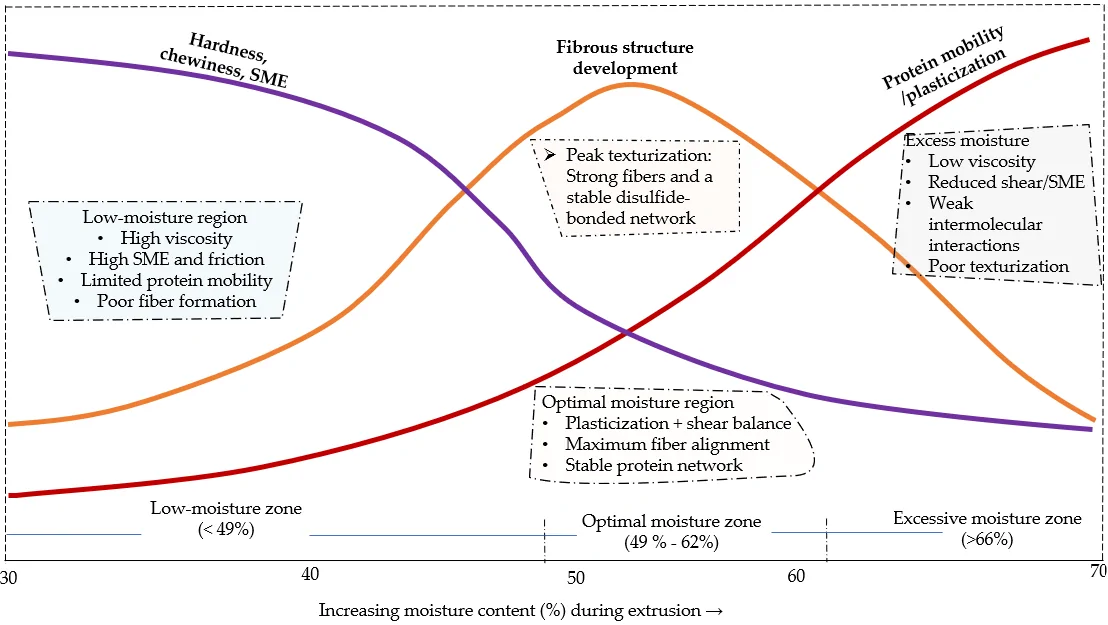
Conceptual trend illustrating the impact of moisture on HME reported in the literature based on Supplementary Table S1. The moisture values (wb%) on the x-axis represent a typical operating range and are intended for illustrative purposes to show qualitative relationships.

**Figure 2:**
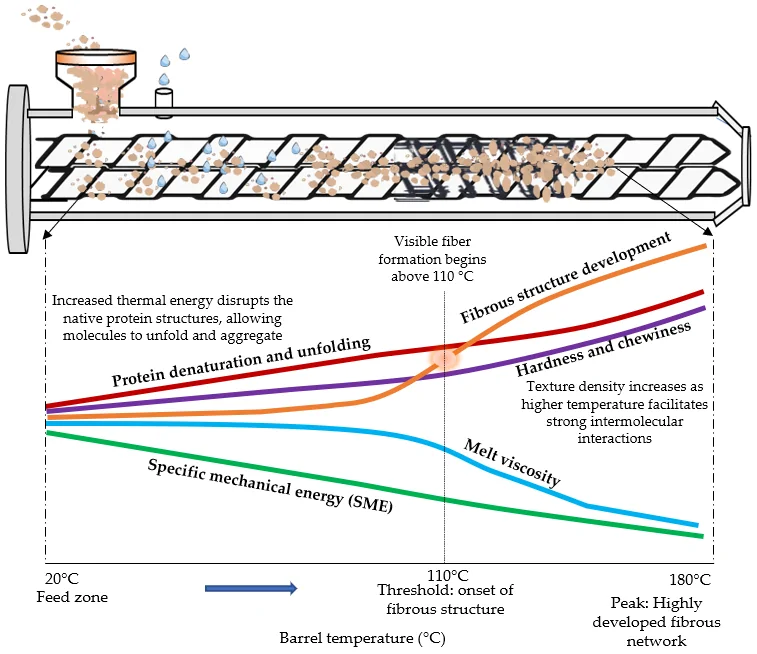
Conceptual trend illustrating the impact of barrel temperature on HME reported in the literature based on Supplementary Table S1. The temperature values on the x-axis represent a typical operating range and are intended for illustrative purposes to show qualitative relationships.

**Figure 3:**
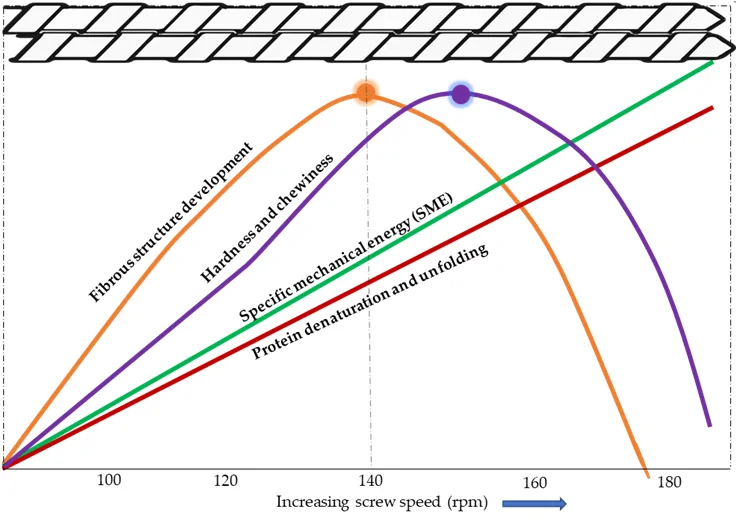
Conceptual trend illustrating the impact of screw speed on HME reported in the literature, based on Supplementary Table S1. The screw speed values on the x-axis represent a typical operating range and are intended for illustrative purposes to show qualitative relationships.

**Figure 4:**
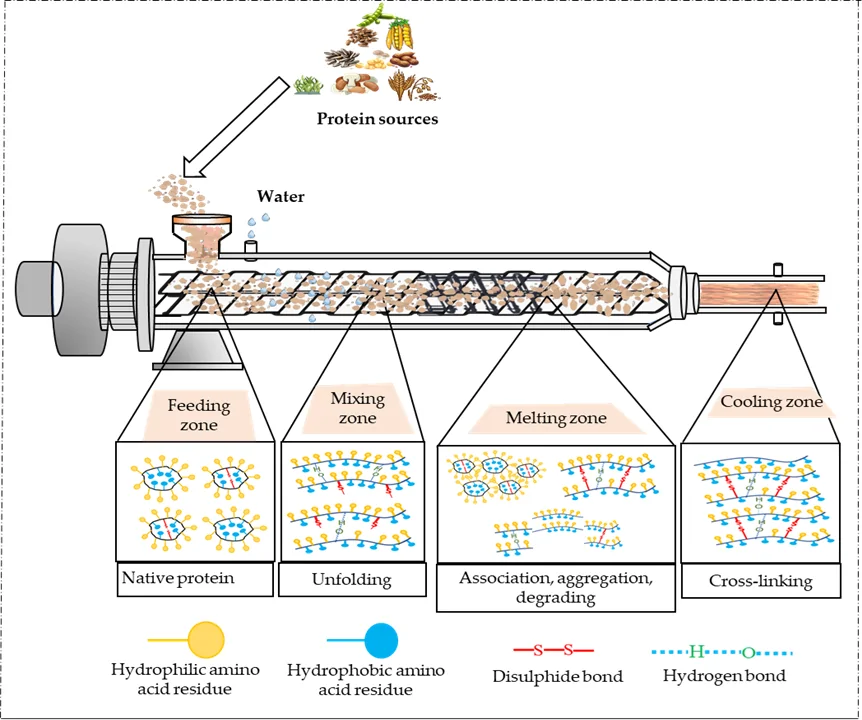
General overview of the HME process for plant protein texturization, illustrating the feeding, mixing, melting, and cooling zones and the corresponding molecular changes, including protein unfolding, intermolecular bonding, network formation, and cross-linking.

**Table 1:** Summary of the impact of screw configuration on HME parameters.

Parameter (Increasing)	Fibrous Structure	Hardness/Chewiness	Mechanical Energy (SME)/Shear	Key Notes	Reference/Condition
Screw configuration (restrictive elements)	General mechanism: Configuration governs mixing efficiency, fill level and energy input. Co-rotating intermeshing twin-screw designs are the most common choice for HME because their mixing, conveying, and shear distribution favor fibrous structure development.				
	—	—	↑ Fill degree up to +379%; SME up ~154%; further +131% when reverse kneading element moved from 150 to 50 mm from the die	Moving the reverse kneading element closer to the die raised material viscosity during extrusion	Reversed helix, staggered elements, narrow kneading discs [89]
	↑ Fiber index improved from 1.01 to 1.27	↑ from 107 to 112 N; chewiness also improved	—	Post-positioned reversing elements showed the strongest fiber formation	Reversing elements and kneading blocks [90]

↑ Strong/consistent increase, and — not addressed in source text.

**Table 2:** Summary of the cooling die effect on HME.

Parameter (Increasing)	Fibrous Structure	Protein Denaturation/Unfolding	Hardness/Chewiness	Mechanical Energy (SME)/Shear	Key Notes	Reference/ Condition
Cooling die temperature/die design	General mechanism: The cooling die controls temperature, morphology and directional alignment as the melt exits the barrel, driving fibrous, anisotropic structure formation through bio-thermo-fluid-dynamic effects on flow behavior and matrix properties.					
	↑ Protein alignment and texturization improved ~6.7%	—	↓ Hardness reduced ~17.5%	—	Higher cooling die temperature resulted in better alignment while compromising hardness	[92] cooling die temperature
	↑↓ Anisotropy and fiber formation increased with cooling temp; higher cooling shear rate negatively affected anisotropy	↑ Post-denaturation aggregation via disulfide bond-mediated cross-linking	↑ Increased via disulfide bond-mediated protein aggregation	—	Higher cooling shear rate had limited effect on hardness but a negative effect on anisotropy	[95]—cooling temp up to 80 °C and shear rate
	↑ Optimal cooling window 30–40 °C; most pronounced alignment at 40 °C	—	—	—	Dynamic elongation flow from the multihole die interacted with cooling temperature	[96]—multihole die, cooling temp
	↑ Flat die promoted densely aligned fibrous structures with improved mechanical strength	—	—	↓ Flat die reduced SME to 669.99 kJ·kg^−1^ by raising die pressure (4.34 MPa) and mass flow rate (299.40 g·min^−1^)	Die geometry also shifted the fractions of bound vs. non-flowing water.	[93]—die geometry (circular/square/flat)

↑ Strong/consistent increase, ↑↓ mixed/optimum exists, ↓ decrease, and — not addressed in source text.

**Table 3:** Various extrusion studies on plant protein-based meat analogues: materials, processing conditions, objectives, and key findings.

Raw Material	Equipment for Extrusion	Objective of the Study	Extrusion Parameters	Findings	References
YP, SPI	Pilot-scale twin-screw extruder (BCTM-30, Buhler, Switzerland) with eight heating blocks and a rectangular cooling die	Investigate the effect of yeast protein on fiber formation, rheological behavior and in vitro digestibility of a soy protein meat analogue produced by HME.	SS: 250 rpm Temperature profile: 25 °C, 30 °C, 45 °C, 60 °C, 80 °C, 110 °C, 140 °C, and 135 °C Cooling zone: 100 °C	Increasing YP (10–40%) improved fiber formation, but 50% YP reduced fiber degree and textural properties (hardness, chewiness, cohesiveness, gumminess), indicating protein network degradation. Higher YP also decreased springiness and cohesiveness, resulting in a weaker, more crumbly structure.	[75]
RP	Co-rotating twin-screw extruder (DS32-III: Jinan Hisense Machinery Co., Ltd.; Jinan, China) SD: 5 mm SL: 742 mm Cooling die: Circular	Investigate the effects of extrusion parameters (screw speed, temperature, and moisture content) on the functional and structural properties of textured rice protein compared with unextruded rice protein.	SS: 100–250 rpm Temperature profile: 90–150 °C MC: 25–40%	Extrusion improved rice protein functionality, increasing solubility (up to 19.76%), water-holding capacity (37.74%), and emulsion stability (152.82%). Extrusion promoted protein aggregation, increased hydrogen bonds, reduced sulfhydryl/disulfide bonds and hydrophobic interactions, and induced secondary structure changes while β-sheets remained dominant.	[98]
SPC, WG, MBP	Twin-screw extruder (Process 11, Thermo Fisher Scientific, Waltham, MA, USA)	Investigate the effects of mung bean protein substitution (0–50%) on the structural, textural, and functional properties of soy protein concentrate–wheat gluten high-moisture extrudates.	SS: 150 rpm Temperature profile: 40, 60, 80, 100, 120, 150, 150 °C Cooling die temperature: 20 Water flow rate: 0.63 kg/h	The fibrous degree increased with MBP addition up to 20% (maximum: 2.42 ± 0.06) due to improved MBP–WG interactions and gel network formation, then decreased at higher MBP levels because of protein network disruption and reduced fibrillation.	[6]
Soy, pea, rice	Co-rotating, intermeshing twin-screw extruder (Clextral BC-21, Firminy, France). Cooling die: Cylindrical	Explore the relationship between rheological properties and structural and textural attributes of soy, pea, and rice protein extrudates and to evaluate the effects of HME on protein interactions, disulfide bonds, and secondary structural changes.	SS: 400 rpm Temperature profile: 20 °C, 50 °C, 80 °C, 110 °C, and 140 °C Cooling zone: 25°C Water flow rate: 3.6 kg/h MC: 60%	Soy and pea protein pastes showed lower tan δ than rice protein, indicating stronger gel structures and higher chewiness after extrusion. Pea protein secondary structures were more affected by extrusion than soy or rice proteins.	[99]
PP, SPI, CP, WG	Isotropic meshing twin-screw extruder (Process 11, Thermo Scientific, Germany) Barrel length: 440 mm Diameter: 11 mm Cooling die: Rectangular	Explore the effects of protein blending (pea, soy, chickpea protein, and wheat gluten) and extrusion on protein structural changes, including secondary structures, disulfide bonds, and surface hydrophobicity, to understand fibrous structure formation in plant-based meat analogues.	SS: 150 rpm Temperature profile: 40 °C, 60 °C, 80 °C, 100 °C, 140 °C, 130 °C and 100 °C Cooling zone: 20 °C Water flow rate: 10.5 mL/min	The PP–CP–WG blend produced extrudates with the highest hardness, chewiness, and fibrous degree. High-moisture extrusion increased disulfide bond formation and β-sheet content while reducing surface hydrophobicity and fluorescence intensity, indicating enhanced protein cross-linking and structural organization.	[33]
PPC, PPI, RP, WG	Co-rotating twin-screw extruder (Process 11 Hygienic, Thermo Scientific, Karlsruhe, Germany) Barrel length: 440 mm SD: 11 mm	Examine the effect of pH conditions on the fibrillation and structure-forming ability of gluten, pea, rice proteins, and pea protein concentrate during high-moisture extrusion and relate raw material properties to extrudate characteristics.	Melt temperature: 95–160 for RP, WG and PPC, 95–135 for PPI Cooling zone: 30 or 45 °C Water flow rate: 194–313 mL/h SS: 200–350 rpm	pH (5 and 7) and temperature effects were studied, and the findings indicate that increasing the raw material pH (to 7) promoted fibrous structure formation at lower extrusion temperatures, resulting in greater protein alignment and tensile strength, demonstrating that pH adjustment enhances plant protein structuring during high-moisture extrusion.	[100]
PPI and maize starch	Parallel twin-screw extruder (Thermo Scientific^TM^, Karlsruhe, Germany) SD: 11 mm L:D: 40:1	Establish relationships between the rheological properties of pea protein isolate–maize starch mixtures and the structural and mechanical properties of high-moisture extrudates under different moisture conditions.	SS: 500 rpm Temperature profile: 60 °C, 80 °C, 100 °C, 120 °C, 130 °C, 130 °C, 110 °C, and 90 °C Cooling zone: 15 or 18 °C MC: 55–65%	Higher moisture (65%) produced more flexible and anisotropic extrudates, whereas lower moisture (55%) resulted in stiffer, more brittle structures. Starch addition softened the extrudates, and early-stage viscoelastic properties strongly correlated with final hardness and anisotropy.	[73]
SPC, WG, PPI, MBPI, CPC, RPI	Co-rotating and intermeshing twin-screw extruder (Process 16 Hygienic, Thermo Fisher Scientific, Germany, SD: 16 mm, L:D: 40:1	Develop plant-based mixed protein matrices (MPMs) using high-moisture extrusion with improved amino acid profiles similar to chicken breast meat and evaluate their structural and textural properties.	SS: 400 rpm Temperature profile: 40, 60, 80, 100, 120, 120, 140, and 140 °C Cooling medium: 80 °C MC: 55 or 60%	Protein source and moisture content influenced extrudate texture, with 50% rice protein producing the highest hardness and soy–pea (55% moisture) or soy–chickpea (60% moisture) blends showing the greatest texturization. Hydrogen bonds and β-sheets were the primary structural stabilizing forces across all formulations.	[101]
Zein protein, potato protein, SPI, starch, high acyl gellan gum, methylcellulose	Co-rotating, intermeshing twin-screw extruder (ZE 12 HMI, Three-Tec GmbH, Seon, Switzerland) SD: 12 mm L:D: 40:1	Develop an alternative low-temperature high-moisture extrusion approach (≤80 °C) using zein, patatin-rich potato protein, pea starch, and high-acyl gellan gum to produce anisotropic meat-like structures through tailored ingredient interactions.	SS: 300 rpm Temperature profile: 40, 50, 60, 70, 70, and 80 °C Cooling die: 50 °C MC: 60% Water flow rate: 0.75 kg/h	A low-temperature extrusion approach (≤80 °C) using zein, potato protein, pea starch, and high-acyl gellan gum produced highly anisotropic structures (anisotropic index up to 1.5). Patatin-rich potato protein and HA gellan gum integrated into the zein network through electrostatic interactions, while pea starch improved water retention and reduced brittleness, enhancing structural formation and processability.	[27]
SPC, WG, zein, rice starch	Co-rotating twin-screw extruder (Evolum 32, Clextral, Firminy, France)	Examine the effects of zein and rice starch incorporation into soy protein-based formulations on the physicochemical, rheological, textural, and microstructural properties of gluten-free high-moisture extrudates.	SS: 500 rpm Water flow rate: 10.5 kg/h Temperature profile: 30, 50, 70, 90, 120, 130, 135, and 135 °C Cooling die: 87 °C	SPC-only extrudates showed the highest hardness, while adding 25% gluten or zein improved fibrous network formation and anisotropy. Rice starch increased water retention, resulting in softer, less fibrous structures. Textural changes were mainly driven by protein network interactions rather than secondary structure alterations.	[102]
SPI, PPI, CPI, MPI	Process 11 twin-screw extruder (Thermo Fisher Scientific Inc., Waltham, MA, USA), SD: 11 mm	Evaluate the effects of different pulse protein sources (soy, pea, chickpea, and mung bean isolates) on the texture, structure, and in vitro digestibility characteristics of high-moisture extrudates.	SS: 150 rpm Temperature profile: 40, 60, 90, 120, 140, 140, 140, and 110 °C Cooling die: 75 °C MC: 60%	High-moisture extrusion (60% moisture) of pulse proteins showed that SPI produced softer, less anisotropic structures, while CPI exhibited the highest anisotropy and digestibility. Higher β-sheet content in SPI contributed to denser structures, and the 7S/11S ratio influenced melt viscosity, anisotropy, and digestibility.	[35]
SPI, SPC, PPI, WG, RPI, wheat starch	Twin-screw extruder (FMHE36-24, FUMACH, Changsha City, China) SD: 36 mm L/D: 24	Examine the changes in water-binding ability of plant proteins from different sources during high-moisture extrusion and evaluate their effects on the water characteristics, distribution, and juiciness of plant-based meat products.	SS: 240 rpm Temperature profile: 60, 80, 145, 150 and 130 °C Cooling die: 70 °C MC: 60%	SPI, SPC, and WG produced centimeter-scale fibrous structures, with SPI showing the most uniform morphology, strongest gel strength, and highest water-holding capacity. SPI extrudates had the lowest water activity and tightly bound water, while RPI showed poor extrusion performance and weak water binding. Protein water-binding ability influenced extrudate juiciness and water distribution.	[103]

MC: Moisture content, SS: screw speed, SD: screw diameter, YP: yeast protein, SPI: soy protein isolate, RP: rice protein, SPC: soy protein concentrate, WG: wheat gluten, MBP: mung bean protein, PP: pea protein, CP: chickpea protein, PPC: pea protein concentrate, PPI: pea protein isolate, MBPI: mung bean protein isolate, CPC: chickpea protein concentrate, MPI: mung bean protein isolate, and RPI: rice protein isolate.

## Data Availability

No new data were created or analyzed in this study. Data sharing is not applicable to this article.

## Supplementary Materials

The following supporting information can be downloaded at https://www.mdpi.com/article/10.3390/foods15193388/s1: Table S1. Impact of barrel temperature, moisture content and screw speed on HME.
