# Supplementary material for: High-Moisture Extrusion of Plant-Based Meat Analogues: Challenges and Opportunities
Source: Foods. 2026 Sep 23;15(19):3388. doi: 10.3390/foods15193388 (PMC13663960; doi:10.3390/foods15193388)
Supplement: Supplementary file 1 [file foods-15-03388-s001.zip › foods-4509602-supplementary.pdf]

Review

High Moisture Extrusion of Plant-Based Meat Analogues: Challenges and Opportunities

Sanjaya Karki[1], Rishabh More[1], Edward Attenborough[2] and Sushil Dhital[1]*

[1]Bioresource Processing Research Institute of Australia (BioPRIA), Department of Chemical and Biological Engineering, Monash University, Clayton, VIC 3800, Australia

[2] Department of Chemical and Biological Engineering, Monash University, Clayton, VIC 3800, Australia*

Correspondence: sushil.dhital@monash.edu

Table S1. Impact of barrel temperature, moisture content and screw speed on HME

Parameter (increasing)	Fibrous structure	Protein denaturation / unfolding	Hardness / chewiness	Mechanical energy (SME) / shear	Key notes	Reference/ Condition
Barrel temperature	General mechanism: Barrel zones progress from ~20–25°C at the feed zone to a 140–160°C melting zone. This melting zone fully denatures the protein, keeps enough viscosity for flow and die filling, and inactivates heat-labile components.					
	↑ Develops above a threshold (~110–130°C)	↑ Melting zone (140–160°C) fully denatures protein structure; higher temperature drives more unfolding, aggregation & intermolecular interaction	↑ Increases with temperature (protein unfolding & aggregation)	—	Threshold varies by protein (110°C for most soy concentrates, 130°C for ALPHA®8); fiber degree kept rising to 180°C for yeast protein [80].	[60, 80 – 82]
Moisture content	General mechanism: Moisture governs protein–water interactions, phase separation, and formation of the thermoplastic protein matrix. Water is a strong plasticizer (small size, low glass transition temperature, hydrogen bonding), stabilizing the protein network; higher moisture lowers viscosity and extrusion resistance, giving larger pores and altered morphology.					
	↑↓ Improves up to ~58–62% moisture, then fiber clarity drops (excessive moisture)	↑↓ Water stabilizes protein conformation & network; both low (30%) and high (60%) extremes caused excessive denaturation [34]	↓ Generally, decreases with increasing moisture	↓ Lower viscosity and extrusion resistance /friction	Optimal texturization window reported anywhere 35–60% depending on protein; Hossain Brishti et al. [34] found optimum at 49.3% — both 30% and 60% caused weaker gels	[34, 61, 77, 80]

Screw speed	General mechanism: Screw speed sets shear rate and material residence time. Higher speed promotes protein deformation, melt development, die pressure build-up and fiber formation, but shortens the residence time available for structure organization — so its effect on final texture depends on where that trade-off lands.					
	↑↓ Well-formed fiber up to 140 rpm, then limited by short residence time at 160 rpm	↑ Higher speed promotes protein deformation/unfolding; low speed + long residence time promoted excessive aggregation instead	↑↓ Increases 100→140 rpm, decreases at 160 rpm	↑ ~60% increase in shear rate at 160 rpm	Lower speeds caused more aggregation, darker color, rougher surfaces (longer residence time)	SPI, 100–160 rpm [84]
	↑ Texturization index improved with screw speed	—	—	—	Protein degradation was limited under the conditions investigated	Hempseed protein [85]
	↓ Degree of texturization decreased ~27% (150→200 rpm at 55% moisture)	—	—	↑ SME increases due to greater shear & friction	Highest SME observed under low feed moisture, high barrel temperature, and high screw speed together	[86]
	—	—	↓ Decreased when screw speed increased 300→400 rpm	—	Consistent with reduced residence time at higher speed	[87]

↑ Strong/consistent increase ↑↓ Mixed / optimum exists ↓ Decrease — Not addressed in source text

Reference:

34. Hossain Brishti, F.; Chay, S.Y.; Muhammad, K.; Rashedi Ismail-Fitry, M.; Zarei, M.; Karthikeyan, S.; Caballero-Briones, F.; Saari, N. Structural and Rheological Changes of Texturized Mung Bean Protein Induced by Feed Moisture during Extrusion. *Food Chem.* **2021**, *344*, doi:10.1016/j.foodchem.2020.128643.

60. Toldrà, M.; Parés, D.; Barnés-Calle, C.; Gou, P.; Fulladosa, E. Techno-Functional and Physico-Chemical Properties of High Moisture Extrudates from Faba Bean (Vicia Faba) Protein Concentrate Using Different Extrusion Conditions. *LWT* **2025**, *225*, doi:10.1016/j.lwt.2025.117898.

61. Elshamy, A.F.; Rosentrater, K.A.; Ghnimi, S.; Hossain, M.K.; Lammers, V.; Heinz, V.; Diakité, M. High-Moisture Meat Analogs Produced from Dry-Fractionated Faba Bean, Yellow Pea, and Functional Soy Proteins: Effects of Mixture Design and Extrusion Parameters on Texture Properties. *Innovative Food Science and Emerging Technologies* **2025**, *100*, doi:10.1016/j.ifset.2025.103927.

77. Hu, A.; Zhou, H.; Guo, F.; Wang, Q.; Zhang, J. Three-Dimensional Porous Fibrous Structural Morphology Changes of High-Moisture Extruded Soy Protein under the Effect of Moisture Content. *Food Hydrocoll.* **2025**, *159*, doi:10.1016/j.foodhyd.2024.110600.

80. Xia, S.; Song, J.; Ma, C.; Hao, T.; Hou, Y.; Shen, S.; Li, K.; Ma, L.; Xue, Y.; Xue, C.; et al. Effects of Moisture Content and Processing Temperature on the Strength and Orientation Regulation of Fibrous Structures in Meat Analogues. *Food Hydrocoll.* **2023**, *145*, doi:10.1016/j.foodhyd.2023.109113.

81. Chew, D.X.; Theng, A.H.P.; Osen, R.; Chiang, J.H. Effects of Different Soy Protein Concentrates on the Structuring Potential and Extrudate Quality of Meat Analogues via High Moisture Extrusion. *Food Structure* **2025**, *45*, doi:10.1016/j.foostr.2025.100448.

82. Högg, E.; Rauh, C. Towards a Better Understanding of Texturization during High-Moisture Extrusion (HME)—Part I: Modeling the Texturability of Plant-Based Proteins. *Foods* **2023**, *12*, doi:10.3390/foods12101955.

84. Sun, D.; Zhou, C.; Yu, H.; Wang, B.; Li, Y.; Wu, M. Integrated Numerical Simulation and Quality Attributes of Soybean Protein Isolate Extrusion under Different Screw Speeds and Combinations. *Innovative Food Science and Emerging Technologies* **2022**, *79*, doi:10.1016/j.ifset.2022.103053.

85. Rajendra, A.; Ying, D.; Warner, R.D.; Ha, M.; Fang, Z. Effect of Extrusion on the Functional, Textural and Colour Characteristics of Texturized Hempseed Protein. *Food Bioproc. Tech.* **2023**, *16*, 98–110, doi:10.1007/s11947-022-02923-z.

86. Maung, T.T.; Gu, B.Y.; Ryu, G.H. Influence of Extrusion Process Parameters on Specific Mechanical Energy and Physical Properties of High-Moisture Meat Analog. *International Journal of Food Engineering* **2021**, *17*, 149–157, doi:10.1515/ijfe-2020-0042.

87. Ribeiro, G.; Piñero, M.Y.; Parle, F.; Blanco, B.; Roman, L. Optimizing Screw Speed and Barrel Temperature for Textural and Nutritional Improvement of Soy-Based High-Moisture Extrudates. *Foods* **2024**, *13*, doi:10.3390/foods13111748.
